# Substance use in adolescence is associated with future cardiovascular disease risk: findings from the national longitudinal study of adolescent to adult health

**DOI:** 10.3389/fnut.2026.1808382

**Published:** 2026-04-15

**Authors:** Prince Nii Ossah Addo, Monique J. Brown, Rajat Das Gupta, Amos Apreku, Casey D. Xavier Hall, Sylvie Naar

**Affiliations:** 1 Center for Translational Behavioral Science, Department of Behavioral Sciences and Social Medicine, College of Medicine, Florida State University, Tallahassee, FL, United States; 2 Department of Epidemiology and Biostatistics, Arnold School of Public Health, University of South Carolina, Columbia, SC, United States; 3 Division of Epidemiology, Department of Medicine, Vanderbilt University Medical Center, Nashville, TN, United States; 4 Department of Biostatistics, T.H. Chan School of Public Health, Harvard University, Boston, MA, United States; 5 Center of Population Sciences for Health Empowerment, College of Nursing, Florida State University, Tallahassee, FL, United States; 6 College of Social Work, Florida State University, Tallahassee, FL, United States

**Keywords:** adolescence, CVD risk, life course epidemiology, risk behavior, substance use

## Abstract

**Background:**

Substance use is a prevalent public health issue among adolescents in the United States (U.S.). While it’s a known risk factor for adult cardiovascular disease (CVD), the long-term impact of adolescent substance use on future CVD risk is understudied. This study aimed to (1) characterize substance use patterns in adolescents and (2) examine their association with future CVD risk.

**Methods:**

We performed a secondary analysis using Waves I and IV of the National Longitudinal Study of Adolescent to Adult Health (Add Health) dataset. All analyses accounted for the Add Health multistage sampling design using survey procedures in SAS. In Wave I, adolescent substance use was evaluated through three self-report measures: binge drinking, marijuana use, and cigarette smoking. Substance use was defined in two ways: first, as mutually exclusive patterns (e.g., smoking only, smoking + binge drinking), and second, by the total number of substances used (0–3) to examine dose–response relationships. CVD risk in adulthood was measured using the 30-year Framingham CVD risk score and categorized as low or high. Using multiple logistic regression, we examined the association between substance use and CVD risk in adulthood, while adjusting for covariates.

**Results:**

The final analytic sample comprised 4,128 participants with an average age of 15 years at baseline. About 26% of adolescents reported binge drinking, and a similar percentage (26%) reported smoking, while 13% used marijuana. Compared with adolescents who reported no substance use, those using one substance had 1.82 times higher odds of CVD in adulthood (95% CI: 1.47–2.25) after controlling for covariates. The odds increased to 2.38 times for two substances (95% CI: 1.74–3.25) and to 2.68 times for three substances (95% CI: 1.98–3.61). Adolescents using multiple substances, like smoking and binge drinking or all three substances, had higher odds of adult CVD compared to non-users.

**Conclusion:**

Substance use in adolescence is strongly associated with elevated CVD risk in adulthood. These findings underscore the need to include substance use prevention and early intervention within broader initiatives to lower the CVD burden. Addressing substance use in young people may thus be a vital opportunity to reduce long-term cardiometabolic risks.

## Introduction

Substance use among adolescents in the United States (U.S.) is a significant public health issue. Data consistently show that experimentation with substances such as tobacco, marijuana, alcohol, and other drugs often begins in adolescence (1, 2). This period is critical in human development, as behavioral patterns formed during adolescence can have a positive or negative impact on future health (3, 4). National data indicate that alcohol, marijuana, and nicotine vaping are the most commonly used substances among U.S. adolescents (5–7).

Research shows that behaviors established early in life, such as substance use, can have enduring effects on cardiovascular health through biological and behavioral mechanisms (8, 9). This supports the life course theory, which posits that early life exposures, including environmental, physical, and psychosocial factors experienced during childhood or adolescence, influence health outcomes later in life (10, 11). Although the immediate risks of adolescent substance use, like poor academic performance, addiction, and injuries, are well understood, its long-term effects on chronic disease development remain underexplored.

Cardiovascular diseases (CVDs) remain the primary cause of death and illness in the United States (12). Risk factors for CVD, such as obesity, hypertension, dyslipidemia, and insulin resistance, often begin early in life (as early as childhood or adolescence) and may continue into adulthood (13, 14). Substance use, especially alcohol, tobacco, and psychoactive drugs like marijuana, has been associated with increased blood pressure, abnormal lipid profiles, and higher systemic inflammation in adults (15–17). However, there is limited long-term longitudinal evidence linking adolescent substance use to CVD risk later in life (18), particularly using nationally representative U.S. samples.

Adolescence is also marked by the onset of other psychosocial and behavioral factors, such as poor sleep health and depressive symptoms (19, 20), which may worsen the cardiometabolic risks associated with substance use. The relationships among these three factors are complex and bidirectional (19, 20). Consequently, sleep health issues and depressive symptoms during adolescence may mediate or confound the association between adolescent substance use and CVD risk later in life.

In this study, we examined the relationship between adolescent substance use and CVD risk in adulthood using a nationally representative, longitudinal sample of adolescents measured at two points, 14 years apart. Specifically, we aimed to [1] characterize substance use patterns among adolescents and [2] assess their associations with subsequent CVD risk. We conducted a sensitivity analysis, adjusting for depressive symptoms and sleep health during adolescence, along with other covariates, due to their complex interactions with substance use and significance for cardiovascular health. By clarifying these long-term relationships, this study enhances the understanding of how early-life behavioral exposures influence adult chronic disease risk and could inform prevention strategies targeting youth health behaviors.

## Materials and methods

### Study sample

We used the publicly available dataset from the National Longitudinal Study of Adolescent to Adult Health (Add Health) for this research. Add Health is a nationally representative study of adolescents in the U.S., sampled from middle and high schools in 1994–1995 and followed into young adulthood (21). The Institutional Review Board (IRB) at the University of North Carolina at Chapel Hill approved the study protocols. We analyzed data from Wave I (1994–1995, ages 12–19) and Wave IV (2008–2009, ages 24–32).

**Figure 1:**
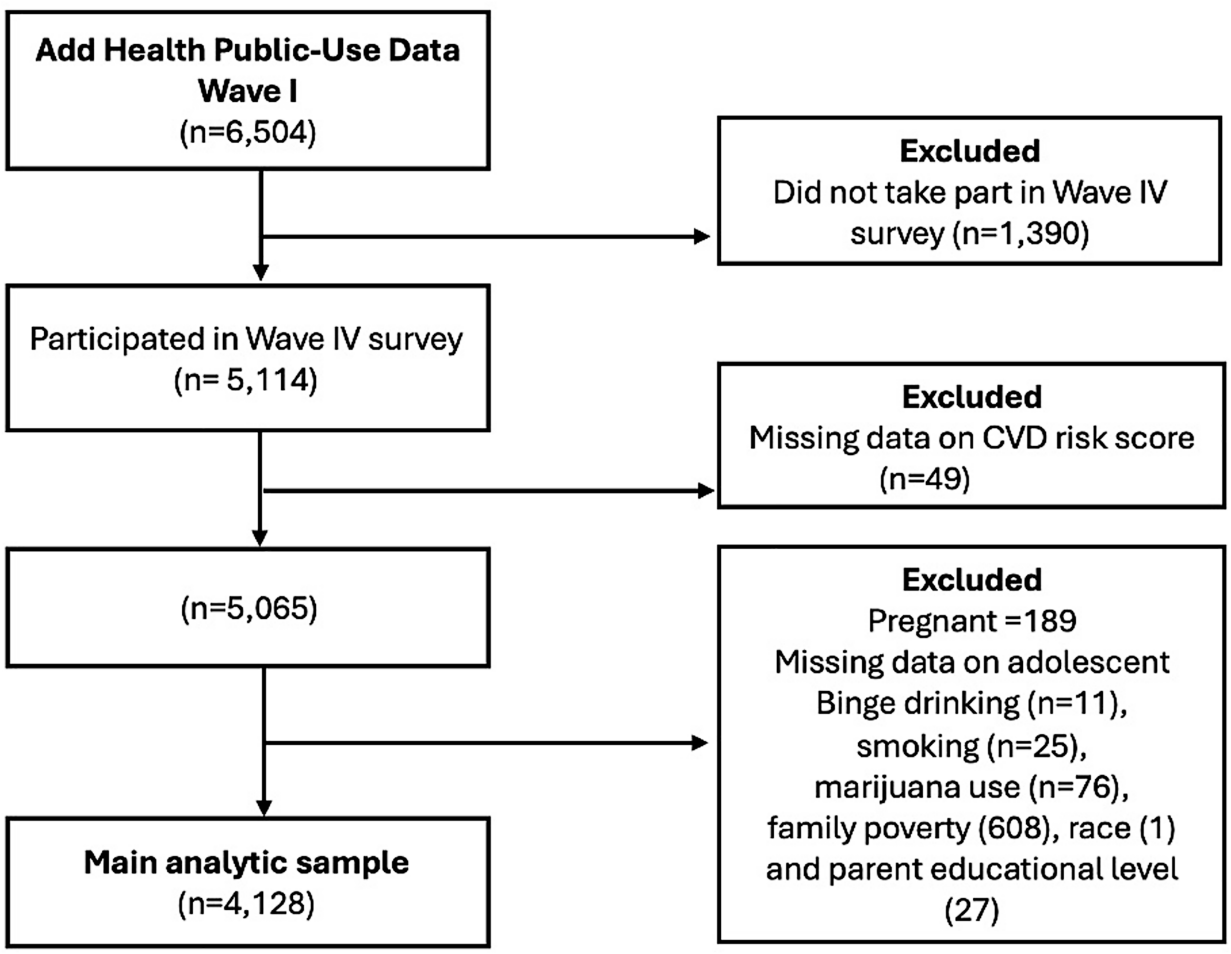
Flow chart showing the final analytic sample used in this study.

Analyses were limited to participants with complete data on CVD risk score, substance use measures, and covariates, as outlined in Figure 1. Pregnant women were excluded from the study. The final analytic sample consisted of 4,128 participants. These participants had backgrounds similar to those of the participants excluded from the study (Supplementary Table 1). Since the data used were publicly available and de-identified, the study was exempt from IRB approval by the University of South Carolina Institutional Review Board.

### Independent variables: substances frequently used by adolescents

Following previous studies that used the Add Health data, substance use was measured with three indicators: smoking, binge drinking, and marijuana use (22). Prior research indicates that participants often accurately report whether they have used substances, but tend to under-report the quantity used. Therefore, substance use was measured as a binary indicator of use (23, 24).

Smoking status was assessed at Wave I with the question: “*During the past 30 days, on how many days did you smoke cigarettes?*” It was then recategorized as “Yes = smoked at least once during the past 30 days” and “No = did not smoke during the past 30 days.”

A single question assessed past-month marijuana use at Wave I: “*During the past 30 days, how many times did you use marijuana?”* It was recategorized as “Yes = used marijuana at least once during the past 30 days” and “No = did not use marijuana.” Similarly, a single question evaluated past-year binge drinking at Wave I: “*Over the past 12 months, on how many days did you drink five or more drinks in a row?*” Binge drinking was recategorized as “Yes = binge drank at least once during the past year” and “No = did not binge drink.”

### Dependent variable: CVD risk score (FRS)

The primary outcome variable, the 30-year CVD risk score, was developed by Pencina and colleagues using data from the Framingham Heart Study (25). The score predicts the likelihood of developing a CVD (congestive heart failure, myocardial infarction, angina pectoris, fatal or non-fatal stroke, and coronary death) within 30 years, while controlling for other causes of death. Risk factors included in the prediction are age, sex, smoking status, diabetes status, use of hypertensive medication, and body mass index. We generated a 30-year CVD risk score for each participant using a SAS macro.^1^ Risk scores ranged from 0 to 100%. Based on earlier research, the score was categorized into low CVD risk (FRS ≤ 20%) and high CVD risk (FRS > 20%) to identify individuals at higher risk of CVD who might benefit from targeted prevention measures (26, 27).

### Covariates

We accounted for factors known from the literature (*a priori*) as potential confounders of the relationship between substance use and CVD. The included baseline socio-demographic and lifestyle factors were age, sex, race, socioeconomic status (measured by parental education and family poverty), and physical activity (8, 26, 27). At baseline, adolescents indicated their participation in moderate-to-vigorous physical activity (MVPA) during the past week. Activity levels were classified as “active” for those with ≥5 MVPA sessions per week and “inactive” for those with fewer than 5 MVPA sessions per week, as per prior research (28, 29).

Sleep health and depressive symptoms were considered as potential confounders in the sensitivity analysis. Sleep health was assessed using three self-reported sleep factors measured at baseline and categorized based on prior research: bedtime (10:00 p.m. or earlier, between 10 p.m. and 11:00 p.m., or after 11:00 p.m./midnight) (30), sleep duration (short, recommended, or long) (31), and insomnia (yes or no) (32). A sleep health score was then calculated by dichotomizing each sleep factor (“long and short sleep duration = 1,” “recommended = 0,” “bedtime after 10 p.m. = 1,” “10 p.m. or earlier = 0,” “insomnia symptoms = 1,” and “no insomnia symptoms = 0”) and summing the scores. Scores ranged from 0 to 3, with higher scores signifying poorer sleep health.

Depressive symptoms were assessed using eight items from the Center for Epidemiological Studies Depression Scale (CES-D) (33). Participants were asked about their feelings of distress (e.g., “felt sad” and “felt depressed”) in the past 7 days, with responses ranging from “0 = never or rarely” to “3 = most of the time or all of the time.” Positive feelings such as “enjoyed life” were reverse-coded. A depression score (ranging from 0 to 24) was created by summing scores from the eight items, with higher scores reflecting greater depressive symptoms.

### Statistical analysis

All analyses accounted for the Add Health stratified, clustered sampling design using survey procedures in SAS (PROC SURVEYLOGISTIC). Sampling weights (Wave IV grand sample cross-sectional weight) and primary sampling units (CLUSTER2) were included in all models to obtain nationally representative estimates, as recommended by Add Health analytic guidelines (34). The analysis employed a survey-domain method, following Add Health’s recommendation, which limits estimates to eligible respondents while keeping the entire sample for accurate variance estimation under the complex survey design. Therefore, eligibility was determined without manually deleting any observations.

We calculated frequencies and percentages for categorical variables, as well as means and standard deviations for continuous variables, for the entire sample and for specific substance-use patterns. Substance use was operationalized in two ways. First, we established mutually exclusive patterns of substance use (none, smoking only, binge drinking only, marijuana only, smoking + binge drinking, smoking + marijuana, binge drinking + marijuana, and all three). Second, to examine dose–response relationships, substance use was measured as the total number of substances used, ranging from 0 to 3. This dose was analyzed both as a categorical variable (0, 1, 2, 3 substances) and as a continuous variable to test for linear trend. We reported both crude and adjusted odds ratios, along with 95% confidence intervals. All analyses were conducted using SAS 9.4.

## Results

The final analytic sample comprised 4,128 adolescents, 21% of whom were identified as having high CVD risk in adulthood. Tables 1 and 2 present the background characteristics of the study participants (n = 4,128) by substance use. The average age of study participants at baseline was 15 years. Most participants were aged 14–17 years (67.7%), male (52.1%), and non-Hispanic White (69.3%). About a quarter (one out of four) of study participants (26%) reported smoking at least once during the past 30 days, and a similar percentage (26%) reported binge drinking over the past 12 months. About 14% of participants used marijuana in the last month (Table 1). Participants who reported only smoking, only binge drinking, and only marijuana use were 8.9, 8.6, and 1.4%, respectively. About 8% reported using all three substances, and 7% reported both smoking and binge drinking (Table 2).

**Table 1:** Adolescent background characteristics by substance use measures.

Characteristics	All	Smoking		*p*-value	Binge drinking		*p*-value	Marijuana use		*p*-value
		Yes	No		Yes	No		Yes	No	
Unweighted (*n*)	4,128	1,024	3,104		1,023	3,105		544	3,584	
Total weighted %	100	26.0	74.0		25.9	74.1		13.9	86.1	
Age, years, %				**<0.001**			**<0.001**			**<0.001**
11–13	18.9	8.9	22.4		6.6	23.2		7.4	20.8	
14–17	67.7	73.8	65.6		71.9	66.3		74.7	66.6	
18–21	13.4	17.2	12.0		21.5	10.5		17.9	12.6	
Sex, %				0.956			**0.001**			0.338
Male	52.1	52.1	52.2		57.5	50.3		54.2	51.8	
Female	47.9	47.9	47.8		42.5	49.7		45.8	48.2	
Race/Ethnicity, %				**<0.001**			**<0.001**			0.809
Hispanic	10.2	8.7	10.7		10.6	10.1		10.0	10.2	
White NH	69.3	78.0	66.2		76.2	66.9		70.1	69.2	
Black NH	15.0	7.3	17.7		8.2	17.3		13.7	15.2	
Other NH	5.5	6.0	5.3		5.7	4.9		6.2	5.4	
Parent education, %				**0.006**			0.380			0.465
Less than high school	14.7	15.7	14.4		15.2	14.6		13.5	15.0	
High school/GED	31.9	33.8	31.2		32.9	31.5		32.1	30.6	
Less than college degree	30.3	31.9	29.7		29.9	31.3		33.4	29.7	
College degree or above	23.1	18.6	24.7		20.6	23.9		22.6	23.2	
Family poverty, %				0.185			0.247			0.510
Yes	22.5	24.8	21.7		20.7	23.1		21.0	22.7	
No	77.5	75.2	78.3		79.3	76.9		79.0	77.3	
Physical activity, %				**0.014**			**0.003**			**<0.001**
Not active	65.3	69.7	63.8		69.4	63.9		74.1	63.9	
Active	34.7	30.3	36.2		30.6	36.1		25.9	36.1	

Bolded *p*-values denote statistical significance.

**Table 2:** Adolescent background characteristics by substance use patterns.

Characteristics	All	Substance use patterns								
		None	Smoking only	Smoking + binge drinking	Smoking + marijuana use	Binge drinking only	Binge drinking + marijuana use	Marijuana use only	All three	*p*-value
Unweighted (*n*)	4,128	2,603	352	287	93	342	102	57	292	
Total weighted %	100	61.6	8.9	7.1	2.3	8.6	2.5	1.4	7.7	
Age, years, %										**<0.001**
11–13	18.9	25.5	13.0	6.0	7.2	6.8	5.3	11.5	7.4	
14–17	67.7	64.6	74.3	68.8	79.2	71.1	69.4	67.1	76.3	
18–21	13.4	9.8	12.8	25.2	13.5	22.1	25.3	21.3	16.3	
Sex, %										**0.001**
Male	52.1	50.2	50.9	55.9	47.1	61.3	67.9	57.5	51.4	
Female	47.9	49.8	49.1	44.1	52.9	38.7	32.1	42.5	48.6	
Race/Ethnicity, %										**<0.001**
Hispanic	10.2	10.4	7.9	10.6	7.0	11.0	16.4	12.6	8.3	
White NH	69.3	65.4	79.2	80.4	63.9	77.0	54.1	60.9	78.8	
Black NH	15.0	18.7	7.1	3.8	18.0	7.8	24.8	22.1	7.4	
Other NH	5.5	5.5	5.8	5.3	11.1	4.2	4.8	4.4	5.5	
Parent education, %										0.188
Less than high school	14.7	14.0	18.7	16.6	15.0	16.7	16.1	15.0	11.9	
High school/GED	31.9	31.5	34.2	37.8	26.8	29.9	33.0	25.9	31.7	
Less than college degree	30.3	29.6	28.9	31.4	38.5	30.0	27.5	33.3	33.8	
College degree or above	23.1	24.9	18.2	14.2	19.7	23.4	23.5	25.8	22.5	
Family poverty, %										**0.034**
Yes	22.5	22.0	31.5	23.1	18.0	18.2	22.9	24.7	20.6	
No	77.7	78.0	68.5	76.9	82.0	81.8	77.1	75.3	79.4	
Physical activity, %										**0.005**
Not active	65.3	63.5	64.1	67.9	74.6	63.8	71.9	64.9	76.3	
Active	34.7	36.5	35.9	32.1	25.4	36.2	28.1	35.1	23.7	

Bolded *p*-values denote statistical significance.

Associations between adolescent substance use and future risk of CVD are presented in Table 3. After controlling for covariates, there was a significant dose–response relationship between substance use and CVD risk. Compared to adolescents who reported no substance use, those using one substance had 1.82 times higher odds of CVD in adulthood (95% CI: 1.47–2.25). The odds increased to 2.38 times for two substances (95% CI: 1.74–3.25) and 2.68 times for three substances (95% CI: 1.98–3.61). This trend was statistically significant (Type 3 *p* < 0.001), with the strength of the association rising consistently as the number of substances used increased. Similar findings were observed when substance use was modeled continuously. Each additional substance used increased the odds of future CVD by 43% (aOR = 1.43, 95% CI: 1.31–1.56, p < 0.001), supporting a significant linear trend.

**Table 3:** Association between substance use (Wave I) and high CVD risk (Wave IV).

Substance use	Crude OR (95% CI)	^a^Adjusted OR (95% CI)
Dose–response (Number of substances)		
None	1.00 (Reference)	1.00 (Reference)
One substance	**2.06 (1.70–2.49)** ^**^	**1.82 (1.47–2.25)** ^**^
Two substances	**2.79 (2.08–3.73)** ^**^	**2.38 (1.74–3.25)** ^**^
Three substances	**2.69 (1.98–3.65)** ^**^	**2.68 (1.98–3.61)** ^**^
Continuous model (per additional substance)		
Per 1-substance increase	**1.49 (1.37–1.63)** ^**^	**1.43 (1.31–1.56)** ^**^
Specific substance use patterns		
None	1.00 (Reference)	1.00 (Reference)
Smoking only	**2.06 (1.54–2.74)** ^**^	**2.11 (1.53–2.89)** ^**^
Smoking + Marijuana use	**2.33 (1.35–4.02)** ^**^	**2.46 (1.40–4.30)** ^**^
Smoking + Binge drinking	**3.63 (2.58–5.12)** ^**^	**3.31 (2.25–4.88)** ^**^
Marijuana only	1.54 (0.75–3.18)	1.26 (0.59–2.66)
Binge drinking only	**2.14 (1.59–2.89)** ^**^	**1.70 (1.21–2.39)** ^**^
Binge drinking + Marijuana use	1.36 (0.75–2.46)	0.86 (0.44–1.67)
All three	**2.69 (1.98–3.65)** ^**^	**2.67 (1.97–3.63)** ^**^

a Adjusted for age, sex, race/ethnicity, parent education, family poverty and physical activity at Wave I.

^*^Indicates *p*-values ≤ 0.05; ^**^Indicates *p*-values ≤ 0.01.

Bolded odds ratios and 95% confidence intervals denote statistical significance.

CVD, Cardiovascular disease; OR, odds ratio; CI, confidence interval.

When analyzing specific substance use patterns, adolescents who only smoked (aOR: 2.11, 95% CI: 1.53–2.89) or only engaged in binge drinking (aOR: 1.70, 95% CI: 1.21–2.39) showed significantly higher odds of developing future CVD compared to non-users. The highest odds were seen in those who both smoked and binge drank (aOR: 3.31, 95% CI: 2.25–4.88), followed by adolescents using all three substances (aOR: 2.67, 95% CI: 1.97–3.63). Smoking combined with marijuana use was also associated with increased odds of future CVD (aOR: 2.46, 95% CI: 1.40–4.30). Conversely, marijuana use alone or together with binge drinking was not significantly associated with CVD risk.

Sensitivity analyses, which included extra adjustment for covariates, resulted in only minor reductions in the effect estimates. However, the direction and statistical significance of the associations remained the same (Supplementary Table 2).

## Discussion

In this study, we explored the association between substance use during adolescence and the risk of CVD in adulthood using a nationally representative longitudinal sample of adolescents. Using multiple substances during adolescence was associated with greater odds of developing CVD later in life, with evidence of a dose–response relationship. Specifically, adolescents who reported smoking combined with binge drinking, smoking with marijuana use, or using all three substances had significantly higher odds of developing adult CVD compared to those who did not use any substances.

The most used substances among adolescents in this study were alcohol (binge drinking) and smoking. This finding is similar to current patterns of substance use among U.S. adolescents reported in national surveys (5, 6), although there has been a noticeable shift from traditional cigarette smoking to nicotine vaping (7). In this study, adolescents who reported smoking alone had the highest odds of developing CVD later in life among those who used only one substance. This finding aligns with published reports of a strong and causal association between early life tobacco smoking and heart disease through damage to the heart and blood vessels (35). Similarly, a nationally representative study reported a higher risk of mortality from heart disease and stroke among participants who started smoking in childhood (36), signifying the long-term impact of early-life smoking on cardiovascular health.

Adolescents who reported binge drinking only had the second-highest odds of future CVD among those who used only one substance. While alcohol affects the adult heart’s normal function, its impact on the adolescent heart is more significant. Binge drinking during adolescence hampers normal heart development, causes physiological hypertrophy (45), and harms vascular function (37). A study by Piano et al. linked binge drinking with several key CVD risk factors in young adults, such as high blood pressure, hypercholesterolemia, and high blood glucose levels, emphasizing the harmful effects of binge drinking on cardiovascular health (38).

Our study found that using multiple substances during adolescence, especially combinations like smoking with binge drinking, smoking with marijuana, or using all three substances, was associated with higher odds of future CVD compared to using a single substance. The odds were greatest among those reporting these dual or triple substance use behaviors. The dose–response relationship between the number of substances used and the risk of CVD observed in this study is consistent with prior research findings (39, 40). For example, Mahtta et al. reported that polysubstance use is associated with elevated risk of developing atherosclerotic CVD (40). Combining multiple substances can produce an additive or even synergistic effect, increasing both the risk and severity of CVD outcomes.

It is important to recognize that other early-life and contextual risk factors may increase adolescents’ likelihood of engaging in substance use and elevate their risk for CVD. For instance, research shows that adverse childhood experiences (ACEs), such as abuse, neglect, and household dysfunction, are associated with earlier initiation and more severe substance use in both adolescence and adulthood (41, 42). These risk factors are also associated with a higher risk of CVD in later life, possibly through long-term neuroendocrine, inflammatory, and behavioral mechanisms (43, 44). Hence, childhood trauma may act as an upstream determinant influencing both substance use and subsequent cardiometabolic health. Although we adjusted for key sociodemographic factors and our main findings stayed consistent in sensitivity analyses, residual confounding from unmeasured early-life adversity or chronic stress might still partly account for the observed associations.

This study’s results have significant implications for prevention and clinical practice. The study findings highlight adolescence as an important period for preventing CVD. Since findings associate adolescent substance use with increased CVD risk later in life, prevention efforts, including risk communication and behavioral interventions, could target adolescents who use substances, especially polysubstance users. Focusing on this group could help stop early behavioral patterns that lead to long-term cardiometabolic issues. The findings also emphasize the importance of including substance use assessments during routine adolescent clinical visits as part of comprehensive CVD risk screening. Doing so allows clinicians to identify youths who may be at higher long-term cardiovascular risk and provide early counseling or referral. Furthermore, adults who used substances during adolescence may need more thorough CVD risk screenings in adulthood, as early-life exposures could influence their risk independently of current behaviors.

### Strengths/limitations

This study’s strengths include assessing how different substance use indicators impact adult CVD risk over 13 years. The use of weighted survey procedures allowed us to obtain nationally representative estimates, making the study findings generalizable to the U.S. population. Our findings highlight early-life substance use as a predictor of future health outcomes. However, the study has some limitations. First, the primary outcome variable, substance use, depended on self-reporting, which may introduce recall bias. Secondly, the sample mainly consisted of non-Hispanic White individuals. Future studies should include more diverse populations to investigate possible variations in associations based on race and ethnicity. Third, some covariates like physical activity probably changed over the 14-year gap between exposures and outcome, potentially influencing CVD risk.

Additionally, some covariates may lie on the causal pathway between substance use and CVD risk (e.g., substance use leading to reduced physical activity, which in turn affects CVD risk). However, we could not establish temporal ordering in this study because we measured both exposure and covariates at the same time point. More longitudinal and mechanistic studies are needed to clarify how early substance use affects biological, behavioral, and social pathways leading to adverse cardiometabolic outcomes. This work will help identify causal mechanisms and guide the development of targeted interventions for at-risk youth. Lastly, since we examined substance use at a single time point, it’s unclear whether this reflects a critical period association, whether enduring substance use patterns over time may be at play, or both. Future research using longitudinally assessed substance use measures should examine whether the observed association between substance use and future CVD is due to a critical exposure period or to cumulative risk.

## Conclusion

Substance use in adolescence is strongly associated with elevated CVD risk in adulthood. These findings underscore the need to include substance use prevention and early intervention within broader initiatives to lower the CVD burden. Addressing substance use in young people may thus be a vital opportunity to reduce long-term cardiometabolic risks.

## Data Availability

Publicly available datasets were analyzed in this study. This data can be found here: https://doi.org/10.3886/ICPSR21600.v26. Further inquiries can be directed to the corresponding author.

## Footnotes

1
https://github.com/zmn0322/30-Year-CVD-Risk-Prediction
