# Supplementary material for: Substance use in adolescence is associated with future cardiovascular disease risk: findings from the national longitudinal study of adolescent to adult health
Source: Front Nutr. 2026 Apr 15;13:1808382. doi: 10.3389/fnut.2026.1808382 (PMC13125064; doi:10.3389/fnut.2026.1808382)
Supplement: Supplementary file 1 [file Table_1.DOCX]

Supplementary Table 1. Characteristics of participants included and excluded

| **Characteristics** | **Included**  **(n=4,128)** | ***Excluded**  **(n=2,376)** |
| --- | --- | --- |
| **Age, years, *M (SD)*** | 15.38 (1.74) | 15.80 (1.84) |
| **Sex, *%*** |  |  |
| Male | 48.00 | 49.18 |
| Female | 52.00 | 50.82 |
| **Race/Ethnicity, *%*** |  |  |
| Hispanic | 9.59 | 14.65 |
| Non-Hispanic White | 61.75 | 50.11 |
| Non-Hispanic Black | 22.84 | 27.06 |
| Other non-Hispanic | 5.81 | 8.19 |
| **Parent Education, *%*** |  |  |
| Less than High School | 13.95 | 19.19 |
| High school/GED | 30.04 | 30.44 |
| Less than college degree | 30.21 | 28.48 |
| College degree or above | 25.80 | 21.89 |
| **Family Poverty, *%*** |  |  |
| Yes | 22.60 | 28.17 |
| No | 77.40 | 71.83 |
| **Smoking Status, %** |  |  |
| Yes | 24.81 | 26.65 |
| No | 75.19 | 73.35 |
| **Binge Drinking, %** |  |  |
| Yes | 24.78 | 27.46 |
| No | 75.22 | 72.54 |
| **Marijuana Use, %** |  |  |
| Yes | 13.18 | 12.77 |
| No | 86.82 | 87.23 |
| **Physical Activity, %** |  |  |
| Not active | 65.75 | 68.82 |
| Active | 34.25 | 31.18 |

*May not sum to the total due to missing values
