# Supplementary material for: Substance use in adolescence is associated with future cardiovascular disease risk: findings from the national longitudinal study of adolescent to adult health
Source: Front Nutr. 2026 Apr 15;13:1808382. doi: 10.3389/fnut.2026.1808382 (PMC13125064; doi:10.3389/fnut.2026.1808382)
Supplement: Supplementary file 2 [file Table_2.DOCX]

Supplementary Table 2. Association between substance use (Wave I) and high CVD risk (Wave IV)

|  | **Crude OR (95% CI)** | **^a^ Adjusted OR (95% CI)** |
| --- | --- | --- |
| **Dose–Response (Number of substances)** |  |  |
| None | 1.00 (Reference) | 1.00 (Reference) |
| One substance | **2.06 (1.70-2.49) **** | **1.77 (1.43–2.20) **** |
| Two substances | **2.79 (2.08-3.73) **** | **2.31 (1.66–3.19) **** |
| Three substances | **2.69 (1.98-3.65) **** | **2.54 (1.89–3.42) **** |
|  |  |  |
| **Continuous model (Per additional substance** |  |  |
| Per 1-substance increase | **1.49 (1.37–1.63) **** | **1.41 (1.28–1.54) **** |
|  |  |  |
| **Substance use patterns** |  |  |
| None | 1.00 (Reference) | 1.00 (Reference) |
| Smoking only | **2.06 (1.54-2.74) **** | **2.06 (1.50-2.82) **** |
| Smoking + Marijuana use | **2.33 (1.35-4.02) **** | **2.38 (1.34-4.15) **** |
| Smoking + Binge drinking | **3.63 (2.58-5.12) **** | **3.22 (2.16-4.78) **** |
| Marijuana only | 1.54 (0.75-3.18) | 1.23 (0.57-2.62) |
| Binge drinking only | **2.14 (1.59-2.89) **** | **1.66 (1.19-2.33) **** |
| Binge drinking + Marijuana use | 1.36 (0.75-2.46) | 0.84 (0.43-1.64) |
| All three | **2.69 (1.98-3.65) **** | **2.55 (1.89-3.44) **** |
| ^a^ Adjusted for age, sex, race/ethnicity, parent education, family poverty, physical activity, depressive symptoms and sleep factors at Wave I  * Indicates P-values ≤ 0.05; ** Indicates P-values ≤ 0.01  Bolded odds ratios and 95% confidence intervals denote statistical significance  CVD: Cardiovascular disease, OR: odds ratio, CI: confidence interval | | |
